# Occupational differences in disability retirement due to a shoulder lesion: do work-related factors matter?

**DOI:** 10.1007/s00420-020-01549-y

**Published:** 2020-05-04

**Authors:** Maria Sirén, Eira Viikari-Juntura, Jari Arokoski, Svetlana Solovieva

**Affiliations:** 1 Department of Physical and Rehabilitation Medicine, Helsinki University Hospital, University of Helsinki, Nordenskiöldinkatu 18 A, 00029 HUS Helsinki, Finland; 2 Finnish Institute of Occupational Health, Helsinki, Finland

**Keywords:** Disability retirement, Occupation, Physical work load factors, Psychosocial factors, Shoulder disease, Work disability

## Abstract

**Objective:**

To identify occupations with a high risk of disability retirement due to a shoulder lesion and to examine the effect of physical and psychosocial work-related factors on occupational differences in disability retirement.

**Methods:**

We followed Finnish wage earners aged 30–59 years (*n* = 1,135,654) from 2005 to 2014 for full disability retirement due to a shoulder lesion. The work-related exposures were assessed with job exposure matrices. We calculated age-adjusted incidence rates and hazard ratios to test for the association between occupation and disability retirement due to a shoulder lesion. We also examined the contribution of work-related exposures to the excess risk of disability retirement.

**Results:**

As compared to professionals, the age-adjusted risk of disability retirement was increased among men in all occupational groups except managers and customer service clerks and among women in several occupational groups. Adjustment for education attenuated the occupational differences considerably, particularly among women. The physical work-related factors fully explained the excess risk of disability retirement due to a shoulder lesion among male finance and sales associate professionals and administrative secretaries as well as among agricultural and fishery workers. In women, the physical work-related factors fully explained the excess risk among construction workers, electricians and plumbers. For both genders, the contribution of psychosocial factors to excess risk of disability retirement was modest and seen for monotonous work only.

**Conclusions:**

A reduction of the level of physical work load factors as well as monotonousness of work has a potential to prevent work disability due to a shoulder lesion.

**Electronic supplementary material:**

The online version of this article (10.1007/s00420-020-01549-y) contains supplementary material, which is available to authorized users.

## Introduction

Population aging is becoming critical over the next decades (World Report on Aging and Heath 2015), especially in the developed countries, challenging sustainable economy. Therefore, lengthening working careers has been set as a national priority in many countries. Musculoskeletal diseases, including shoulder lesions, are the leading causes for work disability in Finland (Pekkala et al. 2018). Disability due to a shoulder lesion is faced at working age, since the incidence of specific shoulder diseases peaks at the years between 45 and 65 years (Greving et al. 2012; van der Windt et al. 1995).

An increased risk of specific shoulder diseases has been found in certain occupational groups, including painters (Loew et al. 2019), farmers, forest workers, construction workers (Rolf et al. 2006), nurses (Chung et al. 2013) and meat-processing workers (Frost and Andersen 1999). These studies have linked the increased risk of shoulder diseases to several exposures. Systematic reviews have provided moderate evidence for the association between shoulder diseases and physical work-related factors (especially arm elevation and shoulder load) and weak evidence for psychosocial work-related factors (van der Molen et al. 2017; van Rijn et al. 2010).

Earlier, we showed that shoulder lesions lead to decreased work participation and preterm exit from paid employment (Siren et al. 2019b). Furthermore, we showed that physically heavy work and working with hands above shoulder level increase the risk of disability retirement due to a shoulder lesion (Siren et al. 2019a). Knowledge on the occupational differences in the risk of disability retirement due to a shoulder lesion and the relative contribution of work-related factors to these differences might help target preventative measures at the workers with the highest risk.

The aims of this study were, first, to identify occupations with a high risk of disability retirement due to a shoulder lesion in the Finnish working population and, second, to examine whether physical and psychosocial work-related factors explain occupational differences in disability retirement.

## Materials and methods

### Setting and data sources

We carried out a longitudinal population-based study, utilizing register data from a 70% random sample of the Finnish population aged 18–70 years living in Finland on 31 December 2004 (~ 2.5 million). Persons aged 30–59 years (as of December 2004), who had gainful job on 1 January 2005, were eligible for the study. We excluded persons, who did not have an occupational title and those who started to receive any retirement-related benefit (full disability retirement, partial or full old-age retirement, or unemployment retirement) before 1 January 2005. Our cohort consisted of 1,135,654 persons (574,617 men and 561,037 women), who were followed from 1 January 2005 till the occurrence of full disability retirement or other pension, death, or end of study period (31 October 2014), whichever came first.

### National register of the Finnish Centre for Pensions (FCP)

Information on employee pensions and earning periods were obtained from the register held by the FCP. The register and work disability pension schemes have been described in detail elsewhere (Gould and Laitinen-Kuikka 2003; Siren et al. 2019b).

### Outcome

The outcome of this study was full time disability retirement (either temporary or permanent) due to a shoulder lesion (ICD-10 code: M75) as the primary diagnosis in the period from 1 January 2005 to 31 October 2014. The FCP register provides information on all disability retirement events with their primary and secondary diagnoses, which are classified according to The International Statistical Classification of Diseases and Related Health Problems, Tenth Revision (ICD-10, Finnish version of ICD-classification 1996).

### Risk factor

Information on persons’ occupation held on the 31 December 2004 was obtained from the Finnish Longitudinal Employer‒Employee Data (FLEED) of Statistics Finland. The occupations were classified at the four-digit level (including a few occupations coded with 5 digits) according to the Classification of Occupations 2001 by Statistics Finland, which is based on the International Standard Classification of Occupations (ISCO-88, https://www.stat.fi/meta/luokitukset/ammatti/001-2001/koko_luokitus_en.html). For the analysis, the occupations were aggregated to two-digit level. The description of occupations at the two-digit level has been reported elsewhere (Solovieva et al. 2018).

### Mediators

Physical work load factors (physically heavy work, manual handling of heavy loads (heavy lifting), working with hands above shoulder level, working in a forward bent posture (forward bent posture) and work demanding high handgrip forces) were estimated with a gender-specific job exposure matrix (JEM) (Solovieva et al. 2012). Psychosocial work-related factors (high job demands, low job control and monotonousness of work) were estimated with a gender-specific JEM (Solovieva et al. 2014b).

The JEMs were developed utilizing information on occupational physical and psychosocial exposures collected via face-to-face interviews using validated questions from a nationally representative population survey (Health 2000 Survey 2008). The matrices cover major physical exposures and psychosocial factors at work in 401 and 365 occupations, respectively (covering more than 80% of all occupations in Finland). Occupations with a small number of respondents and with similar work tasks and exposure profile were grouped (Solovieva et al. 2014a). The physical JEM provides information on the likelihood of exposure (the prevalence of exposure in a specific occupational group). For the analyses, the physical exposures were dichotomized at 0.40 (prevalence of exposure 0–39% classified as non-exposed; ≥ 40% exposed). The psychosocial JEM contains dichotomized exposure measures. Both matrices showed a fairly good validity (Solovieva et al. 2012, 2014).

### Potential confounder

Information on person’s education achieved by the 31 December 2004 was obtained from the Finnish Longitudinal Employer-Employee Data (FLEED) of Statistics Finland. Education was categorized as (1) primary (no education after 9 years of compulsory school and sometimes a voluntary 10th year), (2) secondary (11–12 years of education) and (3) tertiary (13 + years of education).

### Statistical analysis

We calculated age-adjusted (age groups 30–39, 40–49, 50–59 and 60 years) incidence rates (IR per 100,000 person years) of disability retirement due to a shoulder lesion by occupational group and estimated 95% confidence intervals (95% CI) using a Poisson distribution. We calculated the confidence interval for the incidence rate by computing the confidence interval from a sample of observations drawn at random from a Poisson distribution as described by Rothman and Greenland (1998).

We used competing risk regression model (stcrreg, STATA version 14) to estimate hazard ratios (HR) and their 95% CI and to test for the association between occupation and disability retirement due to a shoulder lesion. We accounted for the effect on the outcome of the following competing risks: full disability retirement due to other causes than shoulder lesion, old-age retirement and death. The reference group consisted of professionals.

To quantify the contribution of physical and psychosocial work-related factors to the occupational differences in disability retirement due to a shoulder lesion, we examined, whether the effect of occupation on disability retirement is mediated by those factors.

We assumed that education predetermines the selection of occupation, which in turn predetermines risk factors at work that may cause a shoulder lesion and ultimately result in disability retirement due to this disease. We also assumed that education may be associated with disability retirement directly or indirectly via another pathway than that mentioned above. Since we focused primarily on the contribution of work-related factors, education was considered as a potential confounder in the associations between occupation and disability retirement.

First, we explored the associations between occupation and disability retirement controlling for age (Model 1). After that, we controlled for education (Model 2). The mediating effect of physical and psychosocial work-related factors was tested after the association between occupation and disability retirement was controlled for education. To do this, we included simultaneously into the age and education-adjusted model (Model 2) all physical work load factors (Model 3), all psychosocial work-related factors (Model 4), as well as all work-related (both physical and psychosocial) factors (Model 5).

We also examined separately the contribution of each physical and psychosocial work-related factor to the excess risk of disability retirement. For that, we compared the HR adjusted for age, education and the work-related factor in question with the HR adjusted for age and education (Model 2).

To estimate the contribution of the explanatory factors to the observed statistically significant associations, we calculated the percentage of attenuation of the HR for each occupation (with professionals as reference) after adjustment, using the formula (Hafeman 2009):

$${\text{PRE }}\left( \% \right) = \, \left( {{\text{HRModel}}\_i - {\text{HRModel}}\_i + {1}} \right)/\left( {{\text{ HRModel}}\_i - {1}} \right)*{1}00\% , {\text{PRE}} - {\text{ proportion explained}},\quad i = {1},{ 2}.$$

We used empirical bootstrapping method with 5000 bootstrap samples to estimate 95% confidence intervals (CI) for the percentage explained.

All analyses were made for men and women separately.

## Results

The overall age-adjusted IR of full disability retirement due to a shoulder lesion was higher for men than women, with 36 (95% CI 32–39) and 28 (95% CI 25–31) per 100,000 person years, respectively (Table 1). Occupations with a higher IR than the population average included construction workers, electricians and plumbers (men), service workers (women), agricultural and fishery workers (women), craft workers (women), metal and machinery workers (men), chemical, wood- and metal-processing workers (both genders) and unskilled workers (both genders).

**Table 1:** Age-adjusted incidence rates (IR per 100,000 person years) and 95% confidence intervals (CI) of full disability retirement due to a shoulder lesion during 2005–2014 among 30- to 59-year-old men and women by occupational group

Title	Men				Women			
	*N* total	*N* events	IR	95% CI	*N* total	*N* events	IR	95% CI
*Non-manual workers*								
Managers	33,383	4	2	1–8	15,887	10	11	5–24
Professionals	100,418	20	3	2–6	104,007	16	2	1–5
Physical and engineering science technicians	47,831	40	13	8–23	10,771	6	8	2–25
Environmental officers and nurses	5540	8	22	8–60	42,905	30	10	5–18
Finance and sales associate professionals and administrative secretaries	50,094	38	11	6–19	71,013	43	9	5–14
Office clerks	18,259	40	31	18–52	59,621	50	12	8–20
Customer services clerks	1608	1	6	1–45	18,005	11	8	3–23
Service workers	22,388	53	31	19–55	105,418	300	42	35–52
Shop workers	13,412	26	26	13–52	27,982	78	41	29–58
*Skilled manual workers*								
Agricultural and fishery workers	34,521	116	49	36–68	18,297	66	54	35–88
Construction workers, electricians and plumbers	47,400	301	94	77–114	2066	5	39	12–131
Metal and machinery workers	61,933	265	63	52–79	2958	13	74	35–155
Craft workers	11,618	37	46	26–84	6796	33	72	43–127
Chemical, wood and metal-processing workers	19,024	80	59	41–85	4259	23	74	38–150
Machine operators and assemblers	26,234	88	49	35–69	17,935	68	55	37–85
Professional drivers	45,901	141	46	35–63	2667	3	16	2–113
*Unskilled manual workers*								
Building caretakers, cleaners, assistant nurses and kitchen workers	16,440	74	66	46–97	43,971	265	93	75–116
Unskilled transport, construction and manufacturing workers	18,613	83	66	45–100	6479	37	84	50–144
*All*	574,617	1415	36	32–39	561,037	1057	28	25–31

As compared to professionals, the age-adjusted risk of disability retirement was elevated in all occupations in women and in all occupations except managers and customer services clerks in men (Tables 2, 3, Model 1). Among men, the highest risk of disability retirement was seen for construction workers, electricians and plumbers (HR 32.5, 95% CI 20.7–51.2), followed by unskilled transport, construction and manufacturing workers (HR 23.7, 95% CI 14.5–38.6). Among women, unskilled transport, construction and manufacturing workers had the highest risk of disability retirement (HR 30.9, 95% CI 17.2–55.6) and chemical, wood and metal workers the second highest risk (HR 30.7, 95% CI 16.2–58.1).

**Table 2:** Hazard ratios (HR) and 95% confidence intervals (CI) of full time disability retirement due to a shoulder lesion in 2005–2014 and contribution of education, physical and psychosocial work-related factors to the excess risk of full time disability retirement due to a shoulder lesion in specific occupational groups as compared to professionals 30- to 59-year-old men

Occupational group	Model 1		Model 2				Model 3		Model 4		Model 5	
	HR	95% CI	HR	95% CI	PRE^a^	95% CI	PRE^b^	95% CI	PRE^c^	95% CI	PRE^d^	95% CI
Managers	0.49	0.27–1.45	0.43	0.15–1.25	N/A^e^		N/A		N/A		N/A	
Professionals	1.00		1.00									
Physical and engineering science technicians	4.06	2.37–6.94	2.38	1.39–4.08	54.9	54.5–55.3	17.4	17.1–17.7	3.6	3.5 to 3.8	17.4	17.1–17.7
Environmental officers and nurses	8.33	3.67–18.9	5.14	2.24–11.8	43.5	42.2–44.8	18.4	17.3–19.4	11.8	11.0 to 12.7	19.1	18.0–20.1
Finance and sales associate professionals and administrative secretaries	3.77	2.19–6.47	1.91	1.10–3.30	67.1	66.7–67.6	22.0	21.6–22.3	3.3	3.1 to 3.5	22.0	21.6–22.3
Office clerks	10.8	6.31–18.5	4.52	2.62–7.80	64.1	63.4–64.8	49.4	48.7–50.2	14.8	14.3 to 15.3	52.8	52.1–53.6
Customer services clerks	4.51	0.60–33.6	2.25	0.30–16.8	N/A		N/A		N/A		N/A	
Service workers	16.4	9.81–27.4	6.41	3.78–10.9	64.9	64.2–65.5	52.1	51.5–52.8	6.5	6.1 to 6.8	50.3	49.6–50.9
Shop workers	11.7	6.55–21.0	4.63	2.55–8.39	66.1	65.3–66.9	41.6	40.8–42.4	19.8	19.2 to 20.5	43.3	42.4–44.1
Agricultural and fishery workers	13.4	8.33–21.5	5.03	3.07–8.23	67.5	67.0–68.0	82.6	82.2–83.0	− 5.7^f^	− 6.0 to − 5.4	78.2	77.7–78.6
Construction workers, electricians and plumbers	32.5	20.7–51.2	11.8	7.39–19.0	65.7	65.3–66.1	78.4	78.1–78.8	14.1	13.6 to 14.2	74.5	74.1–74.9
Metal and machinery workers	21.0	13.4–33.2	7.82	4.87–12.6	65.9	65.5–66.3	53.1	52.7–53.5	− 1.5	− 1.6 to − 1.4	47.4	47.0–47.8
Craft workers	16.1	9.35–27.8	6.29	3.61–11.0	65.0	64.1–65.8	52.7	51.8–53.6	13.8	13.2 to 14.4	53.9	53.0–54.8
Chemical, wood- and metal-processing workers	21.6	13.2–35.3	7.87	4.74–13.1	66.7	66.0–67.3	46.1	45.4–46.9	12.2	11.8 to 12.7	49.3	48.6–50.1
Machine operators and assemblers	19.3	11.9–31.4	7.08	4.28–11.7	66.8	66.2–67.3	46.1	45.4–46.7	28.1	27.0 to 28.7	54.3	53.7–54.9
Professional drivers	14.7	9.19–23.4	5.27	3.24–8.57	68.8	68.4–69.3	42.4	41.9–42.8	49.2	48.2 to 49.6	58.5	58.1–59.0
Building caretakers, cleaners, assistant nurses and kitchen workers	20.9	12.8–34.3	7.72	4.63–12.9	66.2	65.5–67.0	54.6	53.9–55.4	6.5	6.7 to 6.9	53.6	52.8–54.3
Unskilled transport, construction and manufacturing workers	23.7	14.5–38.6	8.61	5.20–14.3	66.5	65.8–67.2	64.1	63.4–64.8	30.0	29.6 to 30.6	69.8	69.1–70.4

Model 1: Adjusted for age, Model 2: adjusted for age and education, Model 3: adjusted for age, education and physical work load factors (heavy lifting, working with hands above shoulder level, work demanding high handgrip forces, awkward trunk posture and physically heavy work), Model 4: adjusted for age, education and psychosocial work-related factors (high job demands, low job control and monotonous work), Model 5: adjusted for age, education and physical and psychosocial work-related factors

^a^PRE: percentage explained by education (%)

^b^PRE: percentage explained by physical work load factors (%)

^c^PRE: percentage explained by psychosocial work-related factors (%)

^d^PRE: percentage explained by physical and psychosocial work-related factors (%)

^e^N/A: not applicable

^f^Minus indicates an increase in HR after adjustment

**Table 3:** Hazard ratios (HR) and 95% confidence intervals (CI) of full time disability retirement due to a shoulder lesion in 2005–2014 and contribution of education, physical and psychosocial work-related factors to the excess risk of full time disability retirement due to a shoulder lesion in specific occupational groups as compared to professionals 30- to 59-year-old women

Occupational group	Model 1		Model 2				Model 3		Model 4		Model 5	
	HR	95% CI	HR	95% CI	PRE^a^	95% CI	PRE^b^	95% CI	PRE^c^	95% CI	PRE^d^	95% CI
Managers	3.38	1.53–7.45	2.69	1.23–5.87	29.0	28.3–29.7	2.4	2.1–2.6	1.2	1.0 to 1.4	0.6	0.5–0.7
Professionals	1.00		1.00									
Physical and engineering science technicians	4.01	1.57–10.2	1.49	0.58–3.87	83.7	83.0–84.4	N/A^e^		N/A		N/A	
Environmental officers and nurses	4.83	2.64–8.87	3.60	1.97–6.58	32.1	31.7–32.6	37.3	36.9–37.8	− 3.1^f^	− 3.2 to − 2.9	25.0	24.6–25.4
Finance and sales associate professionals and administrative secretaries	3.59	2.02–6.37	1.44	0.81–2.56	83.0	82.7–83.3	N/A		N/A		N/A	
Office clerks	4.54	2.58–7.97	1.61	0.91–2.87	82.8	82.5–83.1	N/A		N/A		N/A	
Customer services clerks	3.15	1.46–6.79	1.05	0.48–2.29	97.7		N/A		N/A		N/A	
Service workers	16.6	10.0–27.5	4.79	2.83–8.11	75.7	75.4–76.0	50.1	49.8–50.4	5.3	5.1 to 5.4	43.5	43.2–43.8
Shop workers	17.7	10.3–30.3	4.83	2.77–8.42	77.1	76.6–77.6	35.5	34.9–36.1	0.8	0.7 to 0.9	38.4	37.8–39.0
Agricultural and fishery workers	18.9	10.9–32.6	5.47	3.11–9.61	75.0	74.4–75.7	66.9	66.2–67.6	− 7.2	− 7.6 to − 6.8	52.6	51.8–53.3
Construction workers, electricians and plumbers	13.1	4.79–35.7	3.68	1.34–10.1	77.9	76.1–79.6	91.4	90.2–92.6	− 8.6	− 9.9 to − 7.3	76.1	74.3–78.0
Metal and machinery workers	24.6	11.8–50.1	6.78	3.21–14.3	75.5	74.0–77.1	32.4	30.7–34.0	− 2.2	− 2.8 to − 1.7	15.9	14.6–17.2
Craft workers	26.9	14.8–48.9	7.69	4.16–14.2	74.2	73.1–75.2	30.5	29.4–31.6	12.3	11.5 to 13.0	25.1	24.1–26.1
Chemical, wood- and metal-processing workers	30.7	16.2–58.1	8.03	4.17–15.5	76.3	75.1–77.6	44.4	42.9–45.9	36.0	34.5 to 37.4	52.1	50.6–53.6
Machine operators and assemblers	21.3	12.4–36.8	5.57	3.17–9.81	77.5	76.9–78.1	50.1	49.4–50.8	34.1	33.4 to 34.8	51.9	51.1–52.6
Professional drivers	6.02	1.75–20.7	1.65	0.48–5.72	87.1	85.8–88.3	N/A		N/A		N/A	
Building caretakers, cleaners, assistant nurses and kitchen workers	27.2	16.4–45.0	7.25	4.28–12.3	76.1	75.7–76.5	58.2	57.8–58.7	24.0	23.6 to 24.4	54.9	54.4–55.3
Unskilled transport, construction and manufacturing workers	30.9	17.2–55.6	8.00	4.36–14.7	76.6	75.6–77.6	62.6	61.4–63.7	32.4	31.3 to 33.6	63.4	62.3–64.6

Model 1: Adjusted for age; Model 2: adjusted for age and education; Model 3: adjusted for age, education and physical work load factors (heavy lifting, working with hands above shoulder level, work demanding high handgrip forces, awkward trunk posture and physically heavy work); Model 4: adjusted for age, education and psychosocial work-related factors (high job demands, low job control and monotonous work); Model 5: adjusted for age, education and physical and psychosocial work-related factors

^a^PRE: percentage explained by education (%)

^b^PRE: percentage explained by physical work load factors (%)

^c^PRE: percentage explained by psychosocial work-related factors (%)

^d^PRE: percentage explained by physical and psychosocial work-related factors (%)

^e^N/A: not applicable

^f^Minus indicates an increase in HR after adjustment

In both genders, adjustment for education markedly reduced the occupational differences in disability retirement due to a shoulder lesion (Tables 2, 3, Model 2). In men, education explained approximately two-thirds of the risk in most occupations, while in women it explained approximately three-quarters or more of the risk.

Overall, the combined contribution of physical work load factors was higher than the combined contribution of psychosocial work-related factors in both genders (Tables 2, 3, Model 3 and 4). In men, the occupations with very high contribution of physical work load factors were agricultural and fishery workers (82.6%, 95% CI 82.2–83.0) and construction workers, electricians and plumbers (78.4%, 95% CI 78.1–78.8). In women, the highest contribution was observed for construction workers, electricians and plumbers (91.4%, 95% CI 90.2–92.6). The physical work-related factors completely explained the excess risk of disability retirement among male finance and sales associate professionals and administrative secretaries as well as agricultural and fishery workers. Among female construction workers, electricians and plumbers physical work-related factors accounted for all excess risk of disability retirement. Psychosocial factors had a modest effect in male machine operators and assemblers, professional drivers and unskilled transport, construction and manufacturing workers and female chemical, wood- and metal-processing workers as well as unskilled transport, construction and manufacturing workers. Supplementary Tables 1A and Table 1b show the HR and their 95% CI for all models.

A composite exposure, heavy physical work, showed the highest contribution to the excess risk of disability retirement in both genders (Tables 4, 5, Supplementary Tables 2A and Table 2B). The proportion of the risk explained was especially high for male agricultural and fishery workers (86.8%, 95% CI 86.5–87.2) and male and female construction workers, electricians and plumbers (77.8%, 95% CI 77.4–78.2 and 85.1%, 95% CI 83.5–86.6, respectively). Among men, at least 50% of the excess risk of disability retirement was explained by heavy lifting (for agricultural and fishery workers), working with hands above shoulder level (for construction workers, electricians and plumbers) and working in a forward bent posture (for the two above-mentioned occupational groups as well as craft workers). In contrast, none of the specific physical work load factors contributed significantly to the risk of disability retirement among women. Of the psychosocial work-related factors, only monotonous work showed a contribution to the excess risk. This was observed among male machine operators and assemblers, professional drivers and unskilled transport, construction and manufacturing workers and female chemical, wood and metal-processing workers as well as unskilled transport, construction and manufacturing workers.

**Table 4:** Occupational groups with at least 30% of disability retirement due to a shoulder lesion attributable to individual physical or psychosocial work-related factor 30- to 59-year-old men

Occupational groups	Heavy lifting		Hands above shoulder level		High handgrip forces		Forward bent posture		Physically heavy work		High job demands		Low job control		Monotonous work	
	PRE^a^	95% CI	PRE^a^	95% CI	PRE^a^	95% CI	PRE^a^	95% CI	PRE^a^	95% CI	PRE^b^	95% CI	PRE^b^	95% CI	PRE^b^	95% CI
Office clerks	11.9	11.5–12.4	23.0	22.4–23.6	1.4	1.2–1.6	7.4	7.0–7.8	44.0	43.3–44.8	2.3	2.1–2.5	5.1	4.8–5.4	13.9	13.4–14.4
Services workers	17.4	16.9–17.9	1.8	1.7–2.0	2.2	2.0–2.4	22.7	22.3–23.3	51.9	51.3–52.6	0.9	0.8–1.0	2.2	2.0–2.4	5.0	4.7–5.3
Shop workers	33.6	32.8–34.4	18.5	17.8–19.1	10.2	9.7–10.7	25.3	24.6–26.1	43.0	42.1–43.8	19.8	19.2–20.5	5.2	4.9–5.6		
Agricultural and fishery workers	57.3	56.8–57.8	26.3	25.8–26.8	33.5	33.3–34.0	54.6	54.1–55.1	86.8	86.5–87.2			5.0	4.7–5.2	4.5	4.2–4.7
Construction workers, electricians and plumbers	46.7	46.2–47.1	51.4	50.9–51.8	32.1	31.7–32.6	56.2	55.8–56.7	77.8	77.4–78.2	14.2	13.9–14.5	0.6	0.5–0.6		
Metal and machinery workers	25.2	24.9–25.6	34.5	34.1–34.8	27.1	26.8–27.5	48.7	48.3–49.1	52.1	51.7–52.4			4.5	4.4–4.7		
Craft workers	24.8	24.0–25.5	16.8	16.1–17.5	10.4	9.8–11.0	51.4	50.5–52.3	49.3	48.4–50.2			2.3	2.0–2.5	19.3	18.6–20.0
Chemical, wood- and metal-processing workers	31.4	30.8–32.1	14.0	13.5–14.5	16.4	15.9–17.0	35.7	35.0–36.3	48.9	48.2–49.6			4.5	4.2–4.8	20.2	19.7–20.8
Machine operators and assemblers	32.7	32.2–33.3	16.4	16.0–16.9	19.2	18.8–19.7	38.2	37.6–38.7	49.0	48.4–49.6			4.8	4.5–5.0	31.6	31.0–32.1
Professional drivers	32.3	31.9–32.7	6.3	6.1–6.5	12.9	12.6–13.2	13.8	13.5–14.1	48.2	47.8–48.7	17.6	17.2–17.9	4.4	4.3–4.6	36.3	35.9–36.7
Building caretakers, cleaners, assistant nurses and kitchen workers	19.8	19.2–20.4	11.5	11.0–11.9	16.1	15.5–16.6	20.5	19.9–21.2	56.4	55.6–57.2			4.6	4.3–4.9	9.8	9.4–10.3
Unskilled transport, construction and manufacturing workers	43.0	42.3–43.7	18.7	18.1–19.2	21.9	21.4–22.5	34.2	33.5–34.8	81.1	80.5–81.6			4.6	4.3–4.9	33.6	33.0–34.3

^a^PRE—percentage explained (%)—the percentage of attenuation of HR (with professionals as reference) after adjustment for age, education and the physical work load factor in question;

^b^PRE—percentage explained (%)—the percentage of attenuation of HR (with professionals as reference) after adjustment for age, education and the psychosocial factor in question

^c^PRE < 0, indicating an increase in HR after adjustment due to a higher prevalence of exposure in the reference group compared to the occupation in question

**Table 5:** Occupational groups with at least 30% of disability retirement due to a shoulder lesion attributable to individual physical or psychosocial work-related factor 30- to 59-year-old women

Occupational groups	Hands above shoulder level		High handgrip forces		Forward bent posture		Physically heavy work		High job demands		Low job control		Monotonous work	
	PRE^a^	95% CI	PRE^a^	95% CI	PRE^a^	95% CI	PRE^a^	95% CI	PRE^b^	95% CI	PRE^b^	95% CI	PRE^b^	95% CI
Environmental officers and nurses	10.4	10.1–10.7	6.2	5.9–6.4	13.5	13.1–13.8	33.5	33.0–33.9	5.0	4.8–5.2	2.7	2.5–2.8	0.4	0.3–0.4
Services workers	5.3	5.1–5.4	5.0	4.9–5.1	15.0	14.8–15.3	44.1	43.8–44.4			3.2	3.1–3.3	5.5	5.4–5.7
Shop workers	8.9	8.5–9.2	3.1	2.9–3.3	4.7	4.5–4.9	32.1	31.6–32.7						
Agricultural and fishery workers	16.1	15.6–16.6	6.9	6.6–7.3	18.3	17.8–18.9	66.4	65.8–67.1	8.9	8.5–9.4	5.8	5.5–6.2		
Construction workers, electricians and plumbers	41.8	39.7–43.9	17.2	15.5–18.8	29.5	27.5–31.4	85.1	83.5–86.6	9.3	8.1–10.6	6.0	4.9–7.0		
Metal and machinery workers	18.5	17.1–19.9	18.0	16.6–19.4	12.5	11.3–13.6	35.6	33.9–37.4	0.7	0.4–1.0	4.2	3.4–4.9		
Chemical, wood- and metal-processing workers	5.3	4.6–5.9	11.9	11.0–12.9	10.5	9.6–11.4	44.0	42.5–45.4			5.4	4.7–6.1	33.7	32.3–35.1
Machine operators and assemblers	4.6	4.3–4.9	11.8	11.3–12.3	9.8	9.4–10.3	47.0	46.3–47.8	3.9	3.7–4.2	5.7	5.4–6.0	34.1	33.4–34.8
Building caretakers, cleaners, assistant nurses and kitchen workers	15.7	15.3–16.0	9.6	9.3–9.9	15.4	15.0–15.7	47.7	47.2–48.1	3.0	2.9–3.2	5.6	5.4–5.8	24.8	24.4–25.2
Unskilled transport, construction and manufacturing workers	14.3	13.4–15.1	14.7	13.9–15.6	12.6	11.8–13.4	60.7	59.5–61.9	7.4	6.8–8.1	5.4	4.9–6.0	34.0	32.8–35.2

^a^PRE—percentage explained (%)—the percentage of attenuation of HR (with professionals as reference) after adjustment for age, education and the physical work load factor in question;

^b^PRE—percentage explained (%)—the percentage of attenuation of HR (with professionals as reference) after adjustment for age, education and the psychosocial factor in question

^c^PRE < 0, indicating an increase in HR after adjustment due to a higher prevalence of exposure in the reference group compared to the occupation in question. Heavy lifting had a low prevalence among women and was therefore excluded

## Discussion

To our best knowledge, this is the first population-based study on occupational differences in disability retirement due to specific shoulder diseases. In men, the highest incidence rate for disability retirement due to a shoulder lesion was seen in construction workers, electricians and plumbers, followed by unskilled manual workers and metal and machinery workers. In women, the highest and second highest incidence rates were seen in unskilled manual workers. As compared to professionals, the age-adjusted risk of disability retirement was increased among men in all occupational groups except managers and customer service clerks and among women in several occupational groups. Adjustment for education attenuated the occupational differences considerably, particularly among women. The physical work-related factors fully explained the excess risk of disability retirement due to a shoulder lesion among male finance and sales associate professionals and administrative secretaries as well as among agricultural and fishery workers. In women, the physical work-related factors fully explained the excess risk among construction workers, electricians and plumbers. For both genders, the contribution of psychosocial factors to the excess risk of disability retirement was modest and seen for monotonous work only.

There is a growing body of evidence to suggest that shoulder disorders may be increased among some workers (Linaker and Walker-Bone 2015). Previous studies have though reported the incidence or the prevalence of a specific shoulder disease or shoulder pain in selected occupations and have typically provided no gender specific results. In the current study, the outcome was disability retirement due to a shoulder lesion, indicating a more disabling condition. Furthermore, we investigated a broad array of non-manual and manual occupations and quantified the impact of physical and psychosocial work-related factors on the occupational differences separately for the men and women.

Despite the different outcome, our findings are partly in line with the previous studies. For instance, male construction and agricultural workers were over-represented in a German register study on rotator cuff operations (Rolf et al. 2006). Moreover, painters have been reported to have considerably more supraspinatus tears and shoulder pain than controls (Loew et al. 2019) and meat-processing workers to have an increased risk of shoulder impingement syndrome (Frost and Andersen 1999). Furthermore, rotator cuff disease was common in a study on predominantly female nurses (Chung et al. 2013). In line with these results we found a markedly high incidence of disability retirement due to a shoulder lesion among women in the unskilled manual worker group that included assistant nurses as well as in female service workers, including nurses.

Of the physical work-load factors examined in our study, the composite factor of heavy physical work showed the largest contribution to the excess risk of disability retirement due to a shoulder lesion in both genders. Of the specific work load exposures, working with hands above shoulder level and working in a forward bent posture explained at least 50% of the excess risk in male construction workers. Among male agricultural and fishery workers, heavy lifting and forward bent posture were the strongest contributors to the excess risk of disability retirement. For women the contribution of specific work load factors was rather low. Our findings indicate that workplace interventions addressing at least one physical load factor might substantially reduce risk of disability retirement in men, while in women multiple exposures should be targeted to achieve a reduction in disability retirement.

Of psychosocial exposures, monotonous work was the only single exposure that explained a notable proportion of excess risk of disability retirement due to a shoulder lesion. This was seen particularly in manual occupations, such as machine operators and assemblers as well as professional drivers in men, and in chemical, wood- and metal-processing workers in women. Because of the high contribution of psychosocial factors to excess work disability in male professional drivers, workplace interventions targeted at both psychosocial and physical factors would be the most effective.

Considerable occupational differences in disability retirement due to a shoulder lesion remained after controlling for education and physical and psychosocial work-related factors. The risk of disability retirement was still high among unskilled manual workers and most groups of skilled manual workers. Individual factors, such as obesity, may partly explain the remained occupational differences. However, it has been shown for disability retirement due to musculoskeletal diseases that occupational class differences remain even after controlling for occupational and lifestyle factors (Leinonen et al. 2011).

### Strengths and limitations

We used a large nationwide register-based sample representing the Finnish working-age population and followed the persons for nine years. All disability retirement cases were medically certified, and the diagnoses were classified according to ICD-10. Additionally, due to the large sample size, we were able to look at a wide range of occupations for men and women separately. However, some occupational groups were relatively small and had few events, accordingly these results should be interpreted with caution. Furthermore, by utilizing a gender-specific job exposure matrix for the assessment of physical and psychosocial exposures we eliminated the effect of information bias on the observed associations.

Some limitations should be, however, taken into consideration while interpreting the results. We were not able to control for individual factors such as lifestyle factors or illness behavior. In addition, we did not have information on previous occupations, or the length of time the jobs were held. A person with a shoulder problem might have changed his job to a less physically demanding one. This may have led to an underestimation of the occupational differences in disability retirement. Finally, using a job exposure matrix to assess psychosocial factors may have resulted in an underestimation of their contribution (Solovieva et al. 2014a).

## Conclusion

The results of this study suggest that heavy physical work and, in some occupations, also specific exposures, such as working with hands above shoulder level, heavy lifting and working in a forward bent posture, are associated with work disability due to a shoulder lesion. Surveillance of work exposures in high risk occupations could identify workers at risk of work disability due to a shoulder lesion. Reduction of the level of physical work load factors as well as monotonousness of work has a potential to prevent work disability due to a shoulder lesion. In highly exposed occupations, such as skilled and unskilled construction workers as well as agricultural and fishery workers in both genders, and female cleaners, associate nurses and kitchen workers, the potential reduction in disability retirement could be remarkable.

## Electronic supplementary material

Below is the link to the electronic supplementary material.

Supplementary file1 (PDF 145 kb)

Supplementary file2 (PDF 194 kb)
