# Supplementary material for: Occupational differences in disability retirement due to a shoulder lesion: do work-related factors matter?
Source: Int Arch Occup Environ Health. 2020 May 4;93(8):983–93. doi: 10.1007/s00420-020-01549-y (PMC7519916; doi:10.1007/s00420-020-01549-y)
Supplement: Supplementary file 1 — Supplementary file1 (PDF 145 kb) [file 420_2020_1549_MOESM1_ESM.pdf]

Supplementary tables 1A and 1B.

Occupational differences in disability retirement due to a shoulder lesion: do work-related factors matter?

International Archives of Occupational and Environmental Health

Maria Sirén, MD

Helsinki University Hospital, Department of Physical and Rehabilitation Medicine

University of Helsinki, Helsinki, Finland

maria.siren@hus.fi

Eira Viikari-Juntura, MD, PhD

Finnish Institute of Occupational Health, Helsinki, Finland

Jari Arokoski, MD, PhD

Helsinki University Hospital, Department of Physical and Rehabilitation Medicine, Helsinki, Finland

University of Helsinki, Helsinki, Finland

Svetlana Solovieva, PhD

Finnish Institute of Occupational Health, Helsinki, Finland

Supplementary table 1A**.** Hazard ratios (HR) and 95% confidence intervals (CI) of full time disability retirement due to a shoulder lesion in 2005-2014 among 30-60-year-old men by occupational group. Reference group - professionals.

Occupational group	Model 1		Model 2			Model 3			Model 4			Model 5		
	HR	95% CI	HR	95% CI	PRE[1]	HR	95% CI	PRE[2]	HR	95% CI	PRE[3]	HR	95% CI	PRE[4]
Managers	0.49	0.27-1.45	0.43	0.15-1.25	N/A[5]	0.39	0.13-1.15	N/A	0.41	0.14-1.19	N/A	0.39	0.13-1.15	N/A
Professionals	1.00		1.00			1.00			1.00			1.00		
Physical and engineering science technicians	4.06	2.37-6.94	2.38	1.39-4.08	54.9	2.14	1.23-3.70	17.4	2.33	1.36-4.00	3.6	2.14	1.23-3.70	17.4
Environmental officers and nurses	8.33	3.67-18.9	5.14	2.24-11.8	43.5	4.38	1.87-10.2	18.4	4.65	2.02-10.7	11.8	4.35	1.85-10.2	19.1
Finance and sales associate professionals and administrative secretaries	3.77	2.19-6.47	1.91	1.10-3.30	67.1	1.71	0.99-2.97	22.0	1.88	1.08-3.28	3.3	1.71	0.98-3.00	22.0
Office clerks	10.8	6.31-18.5	4.52	2.62-7.80	64.1	2.78	1.57-4.93	49.4	4.00	2.28-7.01	14.8	2.66	1.46-4.80	52.8
Customer services clerks	4.51	0.60-33.6	2.25	0.30-16.8	N/A	2.26	0.30-16.9	N/A	2.23	0.30-16.6	N/A	2.24	0.30-16.7	N/A
Service workers	16.4	9.81-27.4	6.41	3.78-10.9	64.9	3.59	2.04-6.30	52.1	6.06	3.55-10.3	6.5	3.69	2.09-6.51	50.3
Shop workers	11.7	6.55-21.0	4.63	2.55-8.39	66.1	3.12	1.67-5.86	41.6	3.91	2.11-7.24	19.8	3.06	1.60-5.83	43.3
Agricultural and fishery workers	13.4	8.33-21.5	5.03	3.07-8.23	67.5	1.70	0.86-3.33	82.6	5.26	3.14-8.83	-5.7[6]	1.88	0.95-3.74	78.2
Construction workers. electricians and plumbers	32.5	20.7-51.2	11.8	7.39-19.0	65.7	3.33	1.59-6.99	78.4	10.3	6.38-16.5	14.1	3.75	1.76-8.00	74.5
Metal and machinery workers	21.0	13.4-33.2	7.82	4.87-12.6	65.9	4.20	2.27-7.80	53.1	7.92	4.83-13.0	-1.5	4.59	2.45-8.57	47.4
Craft workers	16.1	9.35-27.8	6.29	3.61-11.0	65.0	3.50	1.85-6.60	52.7	5.56	3.13-9.87	13.8	3.44	1.79-6.61	53.9
Chemical. wood and metal processing workers	21.6	13.2-35.3	7.87	4.74-13.1	66.7	4.70	2.64-8.35	46.1	7.03	4.05-12.2	12.2	4.48	2.46-8.16	49.3
Machine operators and assemblers	19.3	11.9-31.4	7.08	4.28-11.7	66.8	4.28	2.40-7.63	46.1	5.37	3.08-9.36	28.1	3.78	2.06-6.93	54.3
Professional drivers	14.7	9.19-23.4	5.27	3.24-8.57	68.8	3.46	2.05-5.84	42.4	3.17	1.84-5.49	49.2	2.77	1.57-4.88	58.5
Building caretakers. cleaners. assistant nurses and kitchen workers	20.9	12.8-34.3	7.72	4.63-12.9	66.2	4.05	2.30-7.13	54.6	7.28	4.25-12.4	6.5	4.12	2.32-7.34	53.6
Unskilled transport. construction and manufacturing workers	23.7	14.5-38.6	8.61	5.20-14.3	66.5	3.73	2.04-6.84	64.1	6.33	3.58-11.2	30.0	3.30	1.75-6.23	69.8

Model 1: Adjusted for age, Model 2: adjusted for age and education, Model 3: adjusted for age, education and physical work load factors (heavy lifting, working with hands above shoulder level, work demanding high handgrip forces, awkward trunk posture and physically heavy work).; Model 4: adjusted for age, education and psychosocial work-related factors (high job demands, low job control and monotonous work).; Model 5: adjusted for age, education and physical and psychosocial work-related factors.
[1]PRE: percentage explained by education (%).[2]PRE: percentage explained by physical work load factors (%);[3]PRE: percentage explained by psychosocial work-related factors (%); [4]PRE: percentage explained by physical and psychosocial work-related factors (%).

[5]N/A: not applicable. [6] Minus indicates an increase in HR after adjustment.

Supplementary table 1B**.** Hazard ratios (HR) and 95% confidence intervals (CI) of full time disability retirement due to a shoulder lesion in 2005-2014 among 30-60-year-old women by occupational group. Reference group - professionals.

Occupational group	Model 1		Model 2			Model 3			Model 4			Model 5		
	HR	95% CI	HR	95% CI	PRE[1]	HR	95% CI	PRE[2]	HR	95% CI	PRE[3]	HR	95% CI	PRE[4]
Managers	3.38	1.53-7.45	2.69	1.23-5.87	29.0	2.65	1.22-5.80	2.4	2.67	1.22-5.87	1.2	2.68	1.22-5.89	0.6
Professionals	1.00		1.00			1.00			1.00			1.00		
Physical and engineering science technicians	4.01	1.57-10.2	1.49	0.58-3.87	83.7	1.53	0.59-3.99	N/A[5]	1.51	0.58-4.00	N/A	1.65	0.63-4.34	N/A
Environmental officers and nurses	4.83	2.64-8.87	3.60	1.97-6.58	32.1	2.63	1.37-5.04	37.3	3.68	2.00-6.78	-3.1[6]	2.95	1.53-5.69	25.0
Finance and sales associate professionals and administrative secretaries	3.59	2.02-6.37	1.44	0.81-2.56	83.0	1.41	0.79-2.51	N/A	1.47	0.81-2.65	N/A	1.55	0.86-2.81	N/A
Office clerks	4.54	2.58-7.97	1.61	0.91-2.87	82.8	1.49	0.84-2.64	N/A	1.55	0.85-2.82	N/A	1.58	0.87-2.88	N/A
Customer services clerks	3.15	1.46-6.79	1.05	0.48-2.29	97.7	1.10	0.50-2.41	N/A	1.01	0.46-2.25	N/A	1.23	0.55-2.76	N/A
Service workers	16.6	10.0-27.5	4.79	2.83-8.11	75.7	2.89	1.61-5.17	50.1	4.59	2.69-7.84	5.3	3.14	1.75-5.61	43.5
Shop workers	17.7	10.3-30.3	4.83	2.77-8.42	77.1	3.47	1.95-6.19	35.5	4.80	2.75-8.37	0.8	3.36	1.89-5.99	38.4
Agricultural and fishery workers	18.9	10.9-32.6	5.47	3.11-9.61	75.0	2.48	1.26-4.86	66.9	5.79	3.11-10.8	-7.2	3.12	1.55-6.32	52.6
Construction workers. electricians and plumbers	13.1	4.79-35.7	3.68	1.34-10.1	77.9	1.23	0.39-3.86	91.4	3.91	1.38-11.0	-8.6	1.64	0.51-5.20	76.1
Metal and machinery workers	24.6	11.8-50.1	6.78	3.21-14.3	75.5	4.91	2.20-10.9	32.4	6.91	3.22-14.8	-2.2	5.86	2.58-13.3	15.9
Craft workers	26.9	14.8-48.9	7.69	4.16-14.2	74.2	5.65	2.91-11.0	30.5	6.87	3.64-13.0	12.3	6.01	3.05-11.8	25.1
Chemical. wood and metal processing workers	30.7	16.2-58.1	8.03	4.17-15.5	76.3	4.91	2.42-9.95	44.4	5.50	2.74-11.1	36.0	4.37	2.13-8.96	52.1
Machine operators and assemblers	21.3	12.4-36.8	5.57	3.17-9.81	77.5	3.28	1.75-6.15	50.1	4.01	2.18-7.39	34.1	3.20	1.69-6.06	51.9
Professional drivers	6.02	1.75-20.7	1.65	0.48-5.72	87.1	1.47	0.42-5.17	N/A	1.52	0.43-5.32	N/A	1.54	0.44-5.44	N/A
Building caretakers. cleaners. assistant nurses and kitchen workers	27.2	16.4-45.0	7.25	4.28-12.3	76.1	3.61	1.97-6.59	58.2	5.75	3.27-10.1	24.0	3.82	2.07-7.04	54.9
Unskilled transport. construction and manufacturing workers	30.9	17.2-55.6	8.00	4.36-14.7	76.6	3.62	1.79-7.32	62.6	5.73	2.97-11.0	32.4	3.56	1.74-7.27	63.4

Model 1: Adjusted for age, Model 2: adjusted for age and education, Model 3: adjusted for age, education and physical work load factors (heavy lifting, working with hands above shoulder level, work demanding high handgrip forces, awkward trunk posture and physically heavy work).; Model 4: adjusted for age, education and psychosocial work-related factors (high job demands, low job control and monotonous work).; Model 5: adjusted for age, education and physical and psychosocial work-related factors.
[1]PRE: percentage explained by education (%).[2]PRE: percentage explained by physical work load factors (%);[3]PRE: percentage explained by psychosocial work-related factors (%); [4]PRE: percentage explained by physical and psychosocial work-related factors (%).

[5]N/A: not applicable. [6] Minus indicates an increase in HR after adjustment
