# Supplementary material for: Occupational differences in disability retirement due to a shoulder lesion: do work-related factors matter?
Source: Int Arch Occup Environ Health. 2020 May 4;93(8):983–93. doi: 10.1007/s00420-020-01549-y (PMC7519916; doi:10.1007/s00420-020-01549-y)
Supplement: Supplementary file 2 — Supplementary file2 (PDF 194 kb) [file 420_2020_1549_MOESM2_ESM.pdf]

Supplementary tables 2A and 2B.

Occupational differences in disability retirement due to a shoulder lesion: do work-related factors matter?

International Archives of Occupational and Environmental Health

Maria Sirén, MD

Helsinki University Hospital, Department of Physical and Rehabilitation Medicine

University of Helsinki, Helsinki, Finland

maria.siren@hus.fi

Eira Viikari-Juntura, MD, PhD

Finnish Institute of Occupational Health, Helsinki, Finland

Jari Arokoski, MD, PhD

Helsinki University Hospital, Department of Physical and Rehabilitation Medicine, Helsinki, Finland

University of Helsinki, Helsinki, Finland

Svetlana Solovieva, PhD

Finnish Institute of Occupational Health, Helsinki, Finland

Supplementary table 2A**.** Age adjusted hazard ratios (HR) of full time disability retirement due to a shoulder lesion in 2005-2014 and contribution of individual physical and psychosocial work-related factors to the excess risk of full time disability retirement due to a shoulder lesion in specific occupational groups as compared to professionals. 30-60-year-old men.

Occupational groups	Model 1	All physical factors	Heavy lifting	Hands above shoulder level	High handgrip forces	Forward bent posture	Physically heavy work	All psychosocial factors	High job demands	Low job control	Monotonous work	All factors
	HR	PRE[1]	PRE[2]	PRE[2]	PRE[2]	PRE[2]	PRE[2]	PRE[3]	PRE[4]	PRE[4]	PRE[4]	PRE[5]
Managers	0.49	N/A[6]	N/A	N/A	N/A	N/A	N/A	N/A	N/A	N/A		N/A
Professionals	1.00											
Physical and engineering science technicians	4.06	17.4	18.8	-3.6[7]	7.2	19.6	21.7	3.6	3.6	1.4	-0.7	17.4
Environmental officers and nurses	8.33	18.4	9.2	7.5	5.1	24.2	16.4	11.8	11.8	0.7	-0.2	19.1
Finance and sales associate professionals and administrative secretaries	3.77	22.0	16.5	-7.7	5.5	5.5	27.5	3.3	3.3	4.4	0.0	22.0
Office clerks	10.8	49.4	11.9	23.0	1.4	7.4	44.0	14.8	2.3	5.1	13.9	52.8
Customer services clerks	4.51	N/A	N/A	N/A	N/A	N/A	N/A	N/A	N/A	N/A	N/A	N/A
Services workers	16.4	52.1	17.4	1.8	2.2	22.7	51.9	6.5	0.9	2.2	5.0	50.3
Shop workers	11.7	41.6	33.6	18.5	10.2	25.3	43.0	19.8	19.8	5.2	-0.6	43.3
Agricultural and fishery workers	13.4	82.6	57.3	26.3	33.5	54.6	86.8	-5.7	-10.7	5.0	4.5	78.2
Construction workers, electricians and plumbers	32.5	78.4	46.7	51.4	32.1	56.2	77.8	14.1	14.2	0.6	-0.9	74.5
Metal and machinery workers	21.0	53.1	25.2	34.5	27.1	48.7	52.1	-1.5	-1.3	4.5	-0.4	47.4
Craft workers	16.1	52.7	24.8	16.8	10.4	51.4	49.3	13.8	-6.0	2.3	19.3	53.9
Chemical, wood and metal processing workers	21.6	46.1	31.4	14.0	16.4	35.7	48.9	12.2	-9.8	4.5	20.2	49.3
Machine operators and assemblers	19.3	46.1	32.7	16.4	19.2	38.2	49.0	28.1	-3.9	4.8	31.6	54.3
Professional drivers	14.7	42.4	32.3	6.3	12.9	13.8	48.2	49.2	17.6	4.4	36.3	58.5
Building caretakers, cleaners, assistant nurses and kitchen workers	20.9	54.6	19.8	11.5	16.1	20.5	56.4	6.5	-5.1	4.6	9.8	53.6
Unskilled transport, construction and manufacturing workers	23.7	64.1	43.0	18.7	21.9	34.2	81.1	30.0	-5.4	4.6	33.6	69.8

Model 1: Adjusted for age.

[1]PRE – percentage explained (%) **-** the percentage of attenuation of HR (with professionals as reference) after adjustment for age, education and all physical work load factor.

[2]PRE – percentage explained (%) **-** the percentage of attenuation of HR (with professionals as reference) after adjustment for age, education and the physical work load factor in question;

[3]PRE – percentage explained (%) **-** the percentage of attenuation of HR (with professionals as reference) after adjustment for age, education and all psychosocial factors.

[4]PRE – percentage explained (%) **-** the percentage of attenuation of HR (with professionals as reference) after adjustment for age, education and the psychosocial factor in question.

[5]PRE – percentage explained (%) **-** the percentage of attenuation of HR (with professionals as reference) after adjustment for age, education and all physical psychosocial work-related factors.

[6]N/A: not applicable; [7] Minus indicates an increase in HR after adjustment.

Supplementary table 2B. Age adjusted hazard ratios (HR) of full time disability retirement due to a shoulder lesion in 2005-2014 and contribution of individual physical and psychosocial work-related factors to the excess risk of full time disability retirement due to a shoulder lesion in specific occupational groups as compared to professionals. 30-60-year-old women.

Occupational groups	Model 1	All physical factors	Heavy lifting	Hands above shoulder level	High handgrip forces	Forward bent posture	Physically heavy work	All psychosocial factors	High job demands	Low job control	Monotonous work	All factors
	HR	PRE[1]	PRE[2]	PRE[2]	PRE[2]	PRE[2]	PRE[2]	PRE[3]	PRE[4]	PRE[4]	PRE[4]	PRE[5]
Managers	3.38	2.4	0.0	-1.8[7]	0.0	-0.6	4.7	1.2	8.3	0.0	4.7	0.6
Professionals	1.00											
Physical and engineering science technicians	4.01	N/A	N/A	N/A	N/A	N/A	N/A	N/A	N/A	N/A	N/A	N/A
Environmental officers and nurses	4.83	37.3	-1.2	10.4	6.2	13.5	33.5	-3.1	5.0	2.7	0.4	25.0
Finance and sales associate professionals and administrative secretaries	3.59	N/A	N/A	N/A	N/A	N/A	N/A	N/A	N/A	N/A	N/A	N/A
Office clerks	4.54	N/A	N/A	N/A	N/A	N/A	N/A	N/A	N/A	N/A	N/A	N/A
Customer services clerks	3.15	N/A	N/A	N/A	N/A	N/A	N/A	N/A	N/A	N/A	N/A	N/A
Services workers	16.6	50.1	-0.8	5.3	5.0	15.0	44.1	5.3	-0.3	3.2	5.5	43.5
Shop workers	17.7	35.5	-0.8	8.9	3.1	4.7	32.1	0.8	-2.6	-0.3	-0.8	38.4
Agricultural and fishery workers	18.9	66.9	-2.5	16.1	6.9	18.3	66.4	-7.2	8.9	5.8	-0.2	52.6
Construction workers, electricians and plumbers	13.1	91.4	-3.7	41.8	17.2	29.5	85.1	-8.6	9.3	6.0	-1.1	76.1
Metal and machinery workers	24.6	32.4	-1.2	18.5	18.0	12.5	35.6	-2.2	0.7	4.2	-0.7	15.9
Craft workers	26.9	30.5	-0.4	1.5	4.0	14.5	26.2	12.3	1.8	4.6	13.0	25.1
Chemical, wood and metal processing workers	30.7	44.4	-1.0	5.3	11.9	10.5	44.0	36.0	-2.6	5.4	33.7	52.1
Machine operators and assemblers	21.3	50.1	-0.7	4.6	11.8	9.8	47.0	34.1	3.9	5.7	34.1	51.9
Professional drivers	6.02	N/A	N/A	N/A	N/A	N/A	N/A	N/A	N/A	N/A	N/A	N/A
Building caretakers, cleaners, assistant nurses and kitchen workers	27.2	58.2	-0.8	15.7	9.6	15.4	47.7	24.0	3.0	5.6	24.8	54.9
Unskilled transport, construction and manufacturing workers	30.9	62.6	-1.6	14.3	14.7	12.6	60.7	32.4	7.4	5.4	34.0	63.4

Model 1: Adjusted for age.

[1]PRE – percentage (%) - the percentage of attenuation of HR (with professionals as reference) after adjustment for age, education and all physical work load factor.

[2]PRE – percentage explained (%) - the percentage of attenuation of HR (with professionals as reference) after adjustment for age, education and the physical work load factor in question;

[3]PRE – percentage explained (%) - the percentage of attenuation of HR (with professionals as reference) after adjustment for age, education and all psychosocial factors.

[4]PRE – percentage explained (%) - the percentage of attenuation of HR (with professionals as reference) after adjustment for age, education and the psychosocial factor in question.

[5]PRE – percentage explained (%) - the percentage of attenuation of HR (with professionals as reference) after adjustment for age, education and all physical psychosocial work-related factors.

[6]N/A: not applicable; [7] Minus indicates an increase in HR after adjustment
